# Advances in Alzheimer’s Disease Drug Development

**DOI:** 10.1186/s12916-015-0297-4

**Published:** 2015-03-25

**Authors:** Michael S Rafii, Paul S Aisen

**Affiliations:** Department of Neurosciences, University of California, 9500 Gilman Dr., MC 0949, La Jolla, San Diego, CA 92093 USA

**Keywords:** Alzheimer’s disease, Beta-amyloid, Clinical trials

## Abstract

Alzheimer’s disease (AD) is the foremost cause of dementia worldwide. Clinically, AD manifests as progressive memory impairment followed by a gradual decline in other cognitive abilities leading to complete functional dependency. Recent biomarker studies indicate that AD is characterized by a long asymptomatic phase, with the development of pathology occurring at least a decade prior to the onset of any symptoms. Current FDA-approved treatments target neurotransmitter abnormalities associated with the disease but do not affect what is believed to be the underlying etiology. In this review, we briefly discuss the most recent therapeutic strategies being employed in AD clinical trials, as well the scientific rationale with which they have been developed.

## Background

Alzheimer’s disease (AD) currently affects over 36 million people worldwide with an estimated global economic impact of approximately $605 billion in 2010 [1] in addition to incalculable social and emotional costs. Initially, AD presents with memory impairment that progressively worsens with concomitant declines in other cognitive abilities and behaviors, which lead to the complete functional dependency that defines the dementia phase of the illness [2]. Converging data from longitudinal biomarker studies indicate that AD is characterized by a long asymptomatic phase, with the initial development of neuropathology beginning approximately 15 to 20 years prior to the onset of any symptoms [3].

## Theories on AD pathogenesis

AD is characterized by the accumulation of neuritic plaques consisting of the β-amyloid (Aβ) peptide and neurofibrillary tangles (NFT) comprised of hyperphosphorylated tau protein. This pathology is associated with disruption of synaptic function leading to neuronal degeneration and brain atrophy. One of the major deterrents to progress in this field is a lack of understanding as to what precisely causes AD. There have been a number of theories proposed to explain the etiology of AD, but to date, no one theory can adequately explain all aspects of this disease. The precise mechanisms in AD progression also remain unclear and there is some controversy regarding the timing of its molecular pathogenesis, including changes in brain amyloid and abnormalities in intracellular tau. Nonetheless, there are three major theories that are presently regarded as most likely to explain the molecular basis of AD and therefore serve as the bases for therapeutic development.

The ‘cholinergic hypothesis’ was the first theory proposed to explain AD and has since led to the development of the only drugs currently approved to treat mild to moderate dementia due to AD [4,5]. This theory is based on the finding that a loss of cholinergic neurons in the Nucleus Basalis of Meynert (NBM), and hence cholinergic activity, is commonly observed in AD brains [6]. Experimental studies in humans and non-human primates suggested a role for acetylcholine in learning and memory [7]. These studies reported that by blocking central cholinergic activity with scopolamine, young subjects would demonstrate memory deficits similar to those seen in aged individuals. These impairments could be reversed by treatment with the cholinergic agonist physostigmine. This theory led to early clinical studies utilizing another type of cholinergic agonist, acetylcholinesterase inhibitors, which showed promise in reversing the memory impairment in AD patients. What specifically leads to the demise of the cholinergic neurons in AD remains unclear and continues to be actively investigated.

There are currently three acetylcholinesterase inhibitors (donepezil, rivastigmine, and galantamine) that have been approved by the Food and Drug Administration (FDA) for the treatment of mild to moderate AD. All of these drugs have been reported to have similar effectiveness; however, donepezil is the most widely prescribed. Clinical trials have reported modest but reproducible improvements in cognition and global functioning by treating patients with donepezil compared to placebo, but these effects were not permanent and patients still demonstrated a decline in cognitive functioning over time [8].

The second and most prevailing theory of AD is the ‘amyloid hypothesis’, which postulates the role of soluble Aβ fragments as synaptotoxic and leading to plaque accumulation and subsequent development of intracellular NFTs. It has been supported by findings that amyloid precursor protein (APP) is located on chromosome 21, which may account for the increased prevalence of dementia in older people with Down syndrome, due to a triplication of this chromosome [9]. Additional support for this hypothesis comes from inherited forms of AD, where mutations have been found within APP leading to autosomal-dominant forms of this disease and from the recently discovered protective effects of an Icelandic mutation (A673T), which leads to a reduction in Aβ formation [10]. Given the pivotal role suggested by the ‘amyloid hypothesis’ for Aβ, it is not surprising that many therapeutics have been designed to interfere with the production of Aβ either through inhibition of enzymes, such as β-secretase, or through strategies aimed at clearing existing plaques within the brain, such as anti-Aβ immunotherapy.

The third hypothesis is the ‘tau hypothesis’, which asserts that abnormalities in the intracellular protein tau are causative. In light of observations that tau oligomers are neurotoxic, clinical symptoms correlate most closely with tau pathology and the fact that anomalous tau hyperphosphorylations constitute a common final pathway for many other dementias, the tau theory is gaining greater acceptance. In this theory, anomalous signaling leads to tau hyperphosphorylation through, for instance, the fyn kinase pathway. Tau modifications lead to its oligomerization and the development of NFTs, resulting in abnormal intracellular trafficking, collapse of the microtubule-based cytoskeleton, and subsequent neuronal demise. As a result of neuronal death, oligomeric forms and tau filaments are released to the extracellular environment, contributing to activation of microglial cells and stimulating the deleterious cycle leading to progressive spread of neuronal degeneration.

Increasing evidence indicates that the tau hypothesis provides close approximation to clinical observations in AD patients. These include the observation that severity of dementia correlates with increasing accumulation of NFTs and level of hyperphosphorylated tau species in the cerebrospinal fluid (CSF) of AD patients correlates with the extent of cognitive impairment [11]. Considering the evidence supporting a neurotoxic role of tau modifications and aggregation in AD, attention has focused during recent years to tau as a target for AD therapies. Recent advances have also been made with radiotracers that permit visualization of tau aggregates [12], along with the development of treatment strategies based on inhibitors of tau modification and/or aggregation.

In this review, we will discuss some of the various therapies being developed based on the major hypotheses.

## Stages of AD

The continuum of AD is now generally considered as having three stages: the ‘preclinical’ stage, in which patients are completely asymptomatic yet harbor AD neuropathology as indicated by biomarkers such as amyloid PET or CSF Aβ abnormalities; the ‘prodromal’ stage, where patients have significant impairment in a single cognitive domain, typically episodic memory; and the more familiar ‘dementia’ stage, in which there are multiple cognitive domains affected with an accompanying functional deficit. The characterization of the preclinical stage of AD, using biomarkers such as amyloid PET, now allows for the earliest point in which treatment can be initiated in AD, and in essence, true secondary prevention. The FDA has provided guidance on developing drugs for preclinical AD, which indicates that an effect on a valid and reliable cognitive assessment used as a single primary efficacy measure may be considered for approval in the context of positive biomarkers of AD [13].

As described, the amyloid hypothesis posits that there is either overproduction of Aβ (i.e., in dominantly inherited forms of AD, Down syndrome) or deficits with mechanisms that clear Aβ from the brain (sporadic form of AD), or possibly both [14]. The excess of Aβ is also thought to lead to hyperphosphorylation of the protein tau within neurons [15]. However, some controversy exists regarding the timing of onset of tau pathology as compared to amyloid-pathology in relation to cognitive decline [16]. Tau is widely-expressed in the brain and binds to and stabilizes microtubules. The hyperphosphorylation of tau disrupts its normal function in regulating axonal transport and leads to the accumulation of neurofibrillary tangles and toxic species of soluble tau [17]. While the exact sequence of molecular events that leads to the development of AD is still unclear, soluble oligomers of Aβ and the resulting formation of amyloid plaques and subsequently neurofibrillary tangles are considered key features of disease pathogenesis. The tight link between all genetic determinants of Aβ production and AD is supportive of the hypothesis and is the central basis for such great focus on anti-amyloid strategies to treat AD.

## The preclinical stage of AD

Subjects in the preclinical AD stage, as defined by biomarkers signaling the presence of amyloid deposition in the brain, are at high risk for progressive cognitive impairment and dementia [18]. Five stages of preclinical AD can now be defined in healthy adults over the age of 65. This classification is based on research criteria developed by the National Institute on Aging and the Alzheimer’s Association, which distinguish three advancing stages (1–3) of preclinical AD [19]. The first stage simply involves the presence of amyloid accumulation in the brain. The second stage is characterized by signs of neuronal injury as evidenced by either regional brain atrophy on volumetric MRI or hypometabolism on brain FDG PET. The third stage involves subtle cognitive impairment that can be quantified in comparison to age- and education-matched controls. Additionally, two other categories exist: Suspected Non-AD Pathophysiology and an Unclassified category [20]. The former is defined by presence of neuronal injury and absence of amyloid with or without signs of cognitive impairment. The Unclassified category is defined by presence of subtle cognitive impairment and absence of amyloid with or without markers of neuronal injury.

## Anti-amyloid strategies

### Passive anti-amyloid immunotherapy

Work from Schenk et al. showed that immunization with Aβ can clear amyloid pathology and lead to cognitive improvements in mouse models of AD [21]. This idea was expanded to show that passive immunization with Aβ antibodies can be similarly effective. Passive immunotherapy remains the leading approach to disease-modifying treatment. These monoclonal antibodies target various domains of the Aβ peptide and prevent aggregation or accelerate removal. Solanezumab is a monoclonal antibody directed against the mid-domain of the Aβ peptide. It recognizes soluble monomeric, not fibrillar (plaque form), Aβ. The therapeutic rationale is that it may exert benefit by sequestering Aβ and removing small soluble species of Aβ that are synaptotoxic. In two Phase III clinical trials, Expedition-1 and -2, 2,052 people with mild to moderate AD were randomized to receive intravenous infusions of 400 mg of solanezumab or placebo once a month for 80 weeks. Data analysis was conducted by the study sponsor and independently by the Alzheimer Disease Cooperative Study (ADCS). Solanezumab was noted to be safe, but treatment showed no improvement on the primary outcome measures of ADAS-Cog11 and ADCS-ADL [22]. However, a pre-specified subgroup analysis of the Expedition-1 trial showed that solanezumab reduced cognitive decline in mild AD when measured by ADAS-Cog 14, prompting the FDA to approve revision of the primary endpoint of Expedition-2 to a single endpoint of cognition in patients with mild AD before the trial database was locked. That analysis saw a trend to improved cognition with solanezumab in people with mild AD, but missed statistical significance. Statistically significant benefit was seen in a pooled analysis of patients with mild AD in both trials [22]. The benefit appeared late, grew over time, and is thought to be consistent with a disease-modifying effect. In mid-2013, Lilly started Expedition-3, a 39-center Phase III trial in 2,100 patients with mild AD and confirmed brain amyloid. Results are expected in late 2016.

Crenezumab is a monoclonal antibody developed by AC Immune and licensed to Genentech. It binds all forms of Aβ, including oligomers and fibrils. In the ABBY study, 122 patients with mild to moderate Alzheimer’s were randomized to receive 300 mg of crenezumab injected subcutaneously every 2 weeks, while 62 volunteers received placebo. Additionally, 163 patients received crenezumab intravenously every 4 weeks and 84 received placebo. No overall difference was noted between the placebo and treatment groups on cognitive co-primary outcome measures. In pre-specified analysis of patients with a Mini-Mental State Examination score of 20 to 26, i.e., the milder subgroup, the higher intravenous dose appeared to slow cognitive decline, but the results did not achieve statistical significance [23].

In the BLAZE study (91 mild to moderate AD patients, Mini-Mental State Examination score of 18 to 26 points with 68 weeks of treatment), which enrolled people with a positive amyloid biomarker, treatment with intravenous crenezumab was also associated with a general trend towards slowing cognitive decline in those with mild disease, although this was not the primary endpoint of the study [23].

Gantenerumab is a monoclonal antibody designed to bind to a conformational epitope on fibrillar Aβ [24]. The therapeutic rationale for this antibody is that it acts centrally to disassemble and degrade amyloid plaques by recruiting microglia and activating phagocytosis. Gantenerumab preferentially interacts with aggregated brain Aβ, both parenchymal and vascular. Roche started a Phase II trial of doses of 105 or 225 mg of gantenerumab injected subcutaneously once a month into 360 participants and in 2012 expanded the study to a Phase II/III registration trial of 770 people. Called SCarlet RoAD, this was a multinational, 159-center study of gantenerumab’s effect on cognition and function in prodromal AD. A futility analysis led to halting the trial in December 2014 due to lack of perceived efficacy as compared to placebo.

Besides removal of Aβ, another approach in AD therapeutic development has been to reduce production of Aβ. The Aβ peptide is cut out of APP by the sequential action of beta- and gamma-secretases. APP is a single-pass membrane protein that is cleaved in the luminal/extracellular region by beta-secretase and within the transmembrane domain by gamma-secretase to release Aβ [25]. Recent genetic evidence shows that mutations near the beta-secretase cleavage site that prevent such cleavages lead to decreased production of Aβ and are, indeed, protective against developing AD dementia, despite ApoE4 status [10].

MK-8931 is a small-molecule inhibitor of beta-secretase cleaving enzyme (BACE)1 and BACE2. The rationale of BACE inhibition is that it represents an upstream interference with the amyloid cascade and reduces cleavage of APP that leads to Aβ production. In April 2012, Phase I data on inhibitor MK-8931 was presented. This drug reduced Aβ CSF levels up to 92% and was well tolerated by patients [26]. In March 2013, data was added from a 1 week trial in 32 mild to moderate AD patients, showing CSF Aβ levels decreased up to 84% [27].

In 2012, Merck launched the EPOCH trial, an 18-month Phase II/III trial comparing 12, 40, or 60 mg/day of MK-8931 given as once-daily tablets to placebo in patients with mild to moderate AD. The trial includes conventional cognitive and functional primary outcomes, as well as sub-studies for biomarker outcomes indicating change in brain amyloid and CSF tau levels, as well as change in brain volume. Further, in 2013, Merck began the APECS trial in 1,500 participants with prodromal AD. These patients have measureable cognitive deficits and a positive PET scan with the newly FDA-approved amyloid tracer flutemetamol, but are not functionally impaired. APECS will compare the 12- and 40-mg once-daily dose to placebo over 24 months.

## Active anti-amyloid immunotherapy

Janssen and Pfizer are conducting Phase II studies to monitor the effects of the Aβ short N-terminus peptide-conjugate vaccine called ACC-001 [28]. AC Immune is continuing a combined Phase I/IIa clinical trial to investigate ACI-24, an active Aβ vaccine intended to induce beta-sheet conformation-specific antibodies, similar to a liposomal vaccine against Aβ1–15 that was previously shown to reduce plaques and restored memory in preclinical studies [29].

Novartis Pharmaceuticals has reported Phase I data for their active Aβ vaccine, CAD106, which consists of multiple copies of Aβ1–6 [30]. This is an active vaccination strategy that aims to elicit a strong antibody response while avoiding inflammatory T cell activation [31]. In 2008, a one-year Phase I trial of two doses of CAD106 in 58 people with mild to moderate AD in Sweden concluded that the vaccine dose-dependently induced Aβ IgG titers that met pre-specified ‘responder’ criteria for an immune response while being generally safe and well-tolerated.

## Preclinical AD trials

As mentioned above, it has been shown that Aβ concentrations in CSF decline decades before the onset of clinical symptoms, which indicates Aβ deposition in brain; and 15 years before clinical symptoms are notable, these Aβ plaques are visible on amyloid PET scans [32]. Thus, intervention has likely to start much earlier and in patients which do not already show symptoms for AD. The design and conduct of preclinical AD trials therefore requires a firm understanding of changes in biomarkers that serve as indicators of AD pathology. Furthermore, there needs to be some translation of the biomarker signal changes into an efficacy signal, as well as clinical meaningfulness. There are now four clinical trials ongoing or soon to be launched for the secondary prevention of AD dementia in the asymptomatic phase of the disease. These are i) Anti-Amyloid in Asymptomatic Alzheimer’s (A4); ii) Dominantly Inherited Alzheimer’s Network- Therapy Unit (DIAN-TU); iii) Alzheimer’s Prevention Initiative - Autosomal Dominant Alzheimer’s Disease –(API-ADAD) trial; and iv) The Alzheimer’s Prevention Initiative (API) ApoE4 trial.

The A4 trial is a multicenter, randomized, double-blind, placebo-controlled, Phase III study comparing 400 mg solanezumab with placebo given as infusions once every 4 weeks over 3 years in approximately 1,000 subjects with preclinical AD, defined as having evidence of elevated brain amyloid pathology before the stage of clinically-evident cognitive impairment.

The DIAN-TU trial will include 160 mutation carriers in their 30s, 40s, or 50s, who range from 15 years before to 10 years after the expected onset of symptoms. Mutation carriers will be randomly assigned to receive one of three investigational drugs or placebo; gantenerumab, solanezumab, and a yet-to-be selected BACE inhibitor, while non-carriers will receive placebo.

The API ADAD trial is studying crenezumab versus placebo in preclinical (asymptomatic) PSEN1 E280A mutation carriers. The API APOE4 trial will test two drugs, an active immunotherapy and an oral BACE inhibitor, and will involve more than 1,300 cognitively-healthy older adults, aged 60 to 75, at high risk of developing symptoms of Alzheimer’s because they are homozygous for apolipoprotein E4. Trial participants will receive the active immunotherapy, the oral medication or a placebo. It is scheduled to begin in the second half of 2015 and last five years.

## Non-anti-amyloid strategies

### Anti-tau strategies

In AD, it is believed that NFTs form later in the disease following Aβ accumulation, while in FTD, no Aβ accumulation is present. Moreover, NFTs seem to most closely track cognitive decline in AD. The current understanding of tau’s function and dysfunction has been aided by the discovery of mutations in the *tau* gene which are responsible for a range of neurodegenerative disorders collectively termed tauopathies, since they are characterized by the presence of aggregated tau deposits. The resultant regional brain atrophy help define the clinical symptomatology observed in each form of tauopathy. However, no mutations in the *tau* gene are associated with AD and the atrophy patterns in tauopathies are as distinct from each other as they are from AD. In AD, there is a medial temporal and bi-parietal atrophy, while in FTD, the most common tauopathy, there is predominantly frontal and temporal atrophy.

Despite its critical role in neurodegenerative disease, therapies targeting tau have not progressed as quickly as those targeting Aβ, likely because tau aggregates are found intra-neuronally, which complicates target engagement [33]. Immunotherapy has recently been harnessed to target tau in mouse models with promising results. Another challenge in tau-based therapeutic research is the lack of understanding as to which forms of tau are neurotoxic. Passive and active immunizations against tau have been analyzed in mice using several different mouse strains, as well as different phospho-tau peptides for active immunizations and anti-tau antibodies for passive immunotherapy, respectively. In the first report on results from immunizations with a 30 amino acid long phosphorylated tau peptide, an effect on the ratios of soluble and insoluble tau, reduction of tangle formation in the immunized mice, as well as functional benefits which were observed in behavior testing for these mice were shown [34].

Interestingly, more than a dozen protein kinases can phosphorylate tau *in vitro* and in neurons. The imbalance in the activities of kinases and phosphatases may also contribute to the aberrant hyperphosphorylation of tau seen in tauopathies. Hence, a provocative therapeutic approach could be to inhibit tau kinases, preventing tau phosphorylation regardless of underlying etiology. Also noteworthy is the recent development of tau PET imaging, including the tracer T807. Ongoing work with such tracers is helping delineate the chronology and regional distribution of tau pathology in various stages of AD, as well as many of the tauopathies.

## Other mechanisms

CERE-110 is a gene therapy product designed to deliver nerve growth factor (NGF). It is composed of an adeno-associated viral vector carrying the gene for NGF and is neurosurgically injected into the NBM. NGF is a neurotrophic protein that can enhance the function of cholinergic neurons in the NBM, prevent their death, and increase production of acetylcholine [35]. A Phase I study has been completed [36] and a multi-center, placebo- controlled Phase II clinical trial in mild to moderate AD conducted by the ADCS is fully enrolled and in the observation phase. Results are expected in mid to late 2015.

The receptor for advanced glycation end-products (RAGE) is involved in the pathogenesis of AD, and that sustained Aβ interaction with RAGE at the blood–brain barrier, or in neuronal or microglial cells, is an important element of amyloid plaque formation and chronic neural dysfunction [37]. TTP488 is a novel, small-molecule, orally active antagonist of RAGE. The ADCS conducted an18-month Phase II trial with 399 subjects with mild to moderate AD. In a pre-specified interim analysis, when 50% of subjects had completed the 6-month visit, the high dose (20 mg) was associated with confusion, falls, and greater ADAS-cog decline, and was discontinued. A second pre-specified analysis compared low-dose (5 mg) and placebo groups for futility and safety approximately 12 months after all subjects were randomized. This analysis met criteria for futility and treatment was discontinued. However, follow-up examination conducted after treatment was suspended did suggest a possible clinical benefit for the low dose [38,39]. A Phase III trial using the 5 mg dose in Mild AD is under consideration.

Encenicline hydrochloride is a partial, selective agonist of the α-7 nicotinic acetylcholine receptor (α7-nAChR) [40]. Cholinergic function declines in Alzheimer’s disease, and currently-approved acetylcholinesterase inhibitor therapies modestly improve cognitive deficits in patients with AD by way of boosting cholinergic transmission. Envivo’s EVP-6124 moved into Phase III for Alzheimer’s. In October 2013, two international Phase III trials began enrolling what are to be 790 patients in each trial with mild to moderate Alzheimer’s who are already taking an acetylcholinesterase inhibitor. The trials will compare two fixed, undisclosed add-on doses of EVP-6124 to placebo, all given as once-daily tablets for 6 months, for cognitive benefit. Called COGNITIV AD, this Phase III program is set to run through 2016.

Pioglitazone is an insulin sensitizer of the thiazolidinedione class of peroxisome-proliferator activated receptor γ (PPAR-γ) agonists. Takeda developed pioglitazone as a once-daily treatment of type 2 diabetes. The PPAR-γ agonist improves cognition in AD mice, and mixed results in prior human trials [41]. In August 2013, Takeda and Zinfandel Pharmaceuticals began ‘Tomorrow,’ a Phase III trial that is to enroll 5,800 cognitively-normal participants and run for 4 years. The study has two separate goals; one is to evaluate how accurately a diagnostic algorithm based on the genes ApoE and TOMM40, developed by Zinfandel, predicts a person’s risk of developing mild cognitive impairment due to AD within 5 years. The other is to evaluate pioglitazone’s ability to delay this diagnosis in people deemed high-risk by the diagnostic assay. The assay is being co-developed as a companion diagnostic for preventive treatment. Participants at high risk of developing mild cognitive impairment due to AD according to the diagnostic algorithm are randomized to pioglitazone or placebo; participants at low risk receive placebo.

Recent evidence suggests that alterations in Fyn, a Src family kinase, might contribute to AD pathogenesis [42]. In addition, Fyn plays a role in the regulation of Aβ production and mediates Aβ-induced synaptic deficits and neurotoxicity [43]. Fyn also induces tyrosine phosphorylation of tau [44]. It is believed that a critical early step in AD pathophysiology is the process by which Aβ interacts with the neuronal surface to trigger downstream pathology, including hyperphosphorylation of tau. Fyn has been implicated in this signaling pathway. A Phase II study of saracatinib (AZD0530), a small molecule inhibitor with high potency for Src and Fyn, for the treatment of AD is currently being conducted by the ADCS.

## Conclusions

Despite the recent negative Phase III clinical trial results for immunotherapeutics in mild to moderate AD, data from trials have raised optimism for anti-amyloid therapies. Specifically, anti-amyloid immunotherapy has now demonstrated its ability to engage CNS Aβ and modify downstream biomarkers (in the case of bapineuzumab) and shown possible cognitive benefit in mild AD (in the case of solanezumab). The entry of potent BACE inhibitors into late stage clinical trials provides additional opportunities to test the ‘amyloid hypothesis’, as do ongoing studies in earlier stages of AD. Enhanced trial methodologies, such as enrichment for preclinical AD (i.e., asymptomatic yet biomarker positive) and composite endpoints with enhanced sensitivity to detect change in early stages of AD, may further improve our capability to demonstrate clinical efficacy. Clinical studies of populations enriched for AD, including autosomal dominant forms of AD, as well as individuals with Down syndrome, will undoubtedly better inform our understanding of early biomarker changes in AD.

As efforts continue towards characterization and diagnosis of AD in its earliest stages, there is growing confidence in the field that we will have a disease-modifying drug that sufficiently engages the appropriate target while adequately assessed in the right phase of the disease, when it will presumably have the greatest efficacy. Tremendous efforts are underway in evaluating different mechanisms of action across the various stages of the AD continuum: preclinical, prodromal, and the dementia phases (Table 1). Progress in understanding the development of tau abnormalities in relation to Aβ deposition and onset of symptoms with new imaging techniques has opened up new avenues for therapeutic research. New compounds targeting tau, including passive immunotherapies, are moving toward human clinical studies. Taken together, these efforts offer the greatest hope of treating this relentless neurodegenerative disease.

**Table 1:** **Selected Alzheimer’s disease drug development programs**

**Drug**	**Mechanism of action**	**Phase of development**	**Stage of disease**
Solanezumab	Aβ – Passive Immunization	III	PRE, MD
Crenezumab	Aβ – Passive Immunization	II	PRE, MD
BIIB-037	Aβ – Passive Immunization	III	PRO, MD
ACI-Anti-Tau Ab	Tau – Passive Immunization	I	MMD
MK-8931	BACE Inhibition	II/III	PRO, MD
ACC-001	Aβ – Active Immunization	II	MMD
ACI-24	Aβ – Active Immunization	II	MMD
CAD-106	Aβ – Active Immunization	II	MMD
ACI-35	Tau – Active Immunization	I	MMD
ACI-24	Tau – Active Immunization	II	MMD
NGF	Neuroprotectant	II	MMD
TTP-488	RAGE Inhibitor	III	MMD
EVP-6124	Cholinergic agonist	II	MMD
AZD0530	Fyn kinase inhibitor	II	MD
Pioglitazone	PPAR-γ agonist	III	PRE

Aβ, Beta-amyloid; BACE, Beta-secretase cleaving enzyme; MD, Mild dementia; MMD, Mild to Moderate Dementia; NGF, Nerve growth factor; PPAR-γ, Peroxisome-proliferator activated receptor γ; PRE, Preclinical; PRO, Prodromal; RAGE, Receptor for advanced glycation end-products.

## Abbreviations

Aβ: β-amyloid
AD: Alzheimer’s disease
ADCS: Alzheimer’s Disease Cooperative Study
APP: amyloid precursor protein
BACE: Beta-secretase cleaving enzyme
CSF: cerebrospinal fluid
FDA: Food and Drug Administration
NBM: Nucleus Basalis of Meynert
NGF: nerve growth factor
NFT: neurofibrillary tangles
PPAR-γ: peroxisome-proliferator activated receptor γ
RAGE: receptor for advanced glycation end-products

## References

[1] Wimo A, Jonsson L, Bond J, Prince M, Winblad B (2013). Alzheimer Disease International. The worldwide economic impact of dementia 2010. Alzheimers Dement.

[2] McKhann G, Drachman D, Folstein M, Katzman R, Price D, Stadlan EM (1984). Clinical diagnosis of Alzheimer’s disease: report of the NINCDS-ADRDAWork Group under the auspices of Department of Health and Human Services Task Force on Alzheimer’s Disease. Neurology..

[3] Jack CR, Knopman DS, Jagust WJ, Petersen RC, Weiner MW, Aisen PS (2013). Tracking pathophysiological processes in Alzheimer’s disease: an updated hypothetical model of dynamic biomarkers. Lancet Neurol..

[4] Bartus RT (2000). On neurodegenerative diseases, models, and treatment strategies: lessons learned and lessons forgotten a generation following the cholinergic hypothesis. Exp Neurol..

[5] Bartus RT, Dean RL, Beer B, Lippa AS (1982). The cholinergic hypothesis of geriatric memory dysfunction. Science..

[6] Davies P, Maloney AJ (1976). Selective loss of central cholinergic neurons in Alzheimer’s disease. Lancet..

[7] Bartus RT (1978). Evidence for a direct cholinergic involvement in the scopolamine induced amnesia in monkeys: effects of concurrent administration of physostigmine and methylphenidate with scopolamine. Pharmacol Biochem Behav..

[8] Doody RS, Dunn JK, Clark CM, Farlow M, Foster NL, Liao T (2001). Chronic donepezil treatment is associated with slowed cognitive decline in Alzheimer’s disease. Dement Geriatr Cogn Disord..

[9] Ness S, Rafii M, Aisen P, Krams M, Silverman W, Manji H (2012). Down’s syndrome and Alzheimer’s disease: towards secondary prevention. Nat Rev Drug Discov..

[10] Jonsson T, Atwal JK, Steinberg S, Snaedal J, Jonsson PV, Bjornsson S (2012). A mutation in APP protects against Alzheimer’s disease and age-related cognitive decline. Nature..

[11] Maccioni RB, Lavados M, Maccioni CB, Mujica C, Bosch R, Farías G (2006). Anomalously phosphorylated tau protein and Abeta fragments in the CSF of Alzheimer’s and MCI subjects. Neurobiol Aging..

[12] Okamura N, Harada R, Furumoto S, Arai H, Yanai K, Kudo Y (2014). Tau PET imaging in Alzheimer’s disease. Curr Neurol Neurosci Rep..

[13] Kozauer N, Katz R (2013). Regulatory innovation and drug development for early-stage Alzheimer’s disease. N Engl J Med..

[14] Selkoe DJ. Alzheimer’s disease. Cold Spring Harb Perspect Biol. 2011;3. doi:10.1101/cshperspect.a004457. 10.1101/cshperspect.a004457 PMC3119915 21576255

[15] Ballatore C, Lee VM, Trojanowski JQ (2007). Tau-mediated neurodegeneration in Alzheimer’s disease and related disorders. Nat Rev Neurosci..

[16] Braak H, Zetterberg H, Del Tredici K, Blennow K (2013). Intraneuronal tau aggregation precedes diffuse plaque deposition, but amyloid-β changes occur before increases of tau in cerebrospinal fluid. Acta Neuropathol..

[17] Iqbal K, Alonso AC, Gong CX, Khatoon S, Pei JJ, Wang JZ (1998). Mechanisms of neurofibrillary degeneration and the formation of neurofibrillary tangles. J Neural Transm..

[18] Rowe CC, Ellis KA, Rimajova M, Bourgeat P, Pike KE, Jones G (2010). Amyloid imaging results from the Australian Imaging, Biomarkers and Lifestyle (AIBL) study of aging. Neurobiol Aging..

[19] Sperling RA, Aisen PS, Beckett LA, Bennett DA, Craft S, Fagan AM (2011). Toward defining the preclinical stages of Alzheimer’s disease: recommendations from the National Institute on Aging-Alzheimer’s Association workgroups on diagnostic guidelines for Alzheimer’s disease. Alzheimers Dement..

[20] Jack CR, Knopman DS, Weigand SD, Wiste HJ, Vemuri P, Lowe V (2012). An operational approach to National Institute on Aging-Alzheimer’s Association criteria for preclinical Alzheimer disease. Ann Neurol..

[21] Schenk D, Barbour R, Dunn W, Gordon G, Grajeda H, Guido T (1999). Immunization with amyloid-beta attenuates Alzheimer-disease-like pathology in the PDAPP mouse. Nature..

[22] Doody RS, Thomas RG, Farlow M, Iwatsubo T, Vellas B, Joffe S (2014). Alzheimer’s Disease Cooperative Study Steering Committee; Solanezumab study group. Phase 3 trials of solanezumab for mild-to-moderate Alzheimer’s disease. N Engl J Med.

[23] Cummings J, Cho W, Ward M, Friesenhahn M, Brunstein F, Honigberg L (2014). A randomized, double-blind, placebo-controlled phase 2 study to evaluate the efficacy and safety of crenezumab in patients with mild to moderate Alzheimer’s disease. Alzheimers Demen.

[24] Bohrmann B, Baumann K, Benz J, Gerber F, Huber W, Knoflach F (2012). Gantenerumab: a novel human anti-beta-amyloid antibody demonstrates sustained cerebral amyloid-β binding and elicits cell-mediated removal of human amyloid-β. J Alzheimers Dis..

[25] Haass C, Selkoe DJ (2007). Soluble protein oligomers in neurodegeneration: lessons from the Alzheimer’s amyloid beta-peptide. Nat Rev Mol Cell Biol..

[26] Forman M, Tseng J, Palcza J, Leempoels J, Ramael S, Krishna G (2012). The novel BACE inhibitor MK-8931 dramatically lowers CSF beta-amyloid peptides in healthy subjects: results from a rising single dose study (PL02. 004). 64th American Academy of Neurology Annual Meeting.

[27] Forman MS PJ, Tseng J, Dockendorf M, Canales C, Apter J, Backonja M, et al. The novel BACe inhibitor MK-8931 dramatically lowers CSF Abeta peptide in patients with mild to moderate Alzheimer’s disease. In: The 11th International Conference on Alzheimer’s and Parkinson’s Disease. Florence, Italy. 2013.

[28] Ryan JM, Grundman M (2009). Anti-amyloid-beta immunotherapy in Alzheimer’s disease: ACC-001 clinical trials are ongoing. J Alzheimer Dis..

[29] Muhs A, Hickman DT, Pihlgren M, Chuard N, Giriens N, Meerschman C (2007). Liposomal vaccines with conformation-specific amyloid antigens define immune response and efficacy in APP transgenic mice. Proc Natl Acad Sci U S A..

[30] Winblad B, Andreasen N, Minthon L, Floesser A, Imbert G, Dumortier T (2012). Safety, tolerability, and antibody response of active beta-amyloid immunotherapy with CAD106 in patients with Alzheimer’s disease: randomized, double-blind, placebo-controlled, first-in-human study. Lancet Neurol..

[31] Lemere CA, Masliah E (2010). Can Alzheimer disease be prevented by amyloid-beta immunotherapy?. Nat Rev Neurol..

[32] Bateman RJ, Xiong C, Benzinger TL, Fagan AM, Goate A, Fox NC (2012). Dominantly Inherited Alzheimer Network Clinical and biomarker changes in dominantly inherited Alzheimer’s disease. N Engl J Med..

[33] Sigurdsson EM (2009). Tau-focused immunotherapy for Alzheimer’s disease and related tauopathies. Curr Alzheimer Res..

[34] Asuni AA, Boutajangout A, Quartermain D, Sigurdsson EM (2007). Immunotherapy targeting pathological tau conformers in a tangle mouse model reduces brain pathology with associated functional improvements. J Neurosci..

[35] Hefti F (1986). Nerve growth factor promotes survival of septal cholinergic neurons after fimbrial transections. J Neurosci..

[36] Rafii MS, Baumann TL, Bakay RA, Ostrove JM, Siffert J, Fleisher AS (2014). A phase 1 study of stereotactic gene delivery of AAV2-NGF for Alzheimer’s disease. Alzheimers Dement..

[37] Schmidt AM, Sahagan B, Nelson RB, Selmer J, Rothlein R, Bell JM (2009). The role of RAGE in amyloid-beta peptide-mediated pathology in Alzheimer’s disease. Curr Opin Investig Drugs..

[38] Galasko D, Bell J, Mancuso JY, Kupiec JW, Sabbagh MN, van Dyck C (2014). Clinical trial of an inhibitor of RAGE-beta-amyloid interactions in Alzheimer disease. Neurology..

[39] Burstein AH, Grimes I, Galasko DR, Aisen PS, Sabbagh M, Mjalli AM (2014). Effect of TTP488 in patients with mild to moderate Alzheimer’s disease. BMC Neurol..

[40] Prickaerts J, van Goethem NP, Chesworth R, Shapiro G, Boess FG, Methfessel C (2012). EVP-6124, a novel and selective α7 nicotinic acetylcholine receptor partial agonist, improves memory performance by potentiating the acetylcholine response of α7 nicotinic acetylcholine receptors. Neuropharmacology..

[41] Miller BW, Willett KC, Desilets AR (2011). Rosiglitazone and pioglitazone for the treatment of Alzheimer’s disease. Ann Pharmacother..

[42] Shirazi SK, Wood JG (1993). The protein tyrosine kinase, Fyn, in Alzheimer’s disease pathology. Neuroreport..

[43] Chin J, Palop JJ, Yu GQ, Kojima N, Masliah E, Mucke L (2004). Fyn kinase modulates synaptotoxicity, but not aberrant sprouting, in human amyloid precursor protein transgenic mice. J Neurosci..

[44] Lee G, Thangavel R, Sharma VM, Litersky JM, Bhaskar K, Fang SM (2004). Phosphorylation of tau by Fyn: implications for Alzheimer’s disease. J Neurosci..

